# Supplementary material for: Future Indian Ocean warming patterns
Source: Nat Commun. 2023 Mar 30;14:1789. doi: 10.1038/s41467-023-37435-7 (PMC10063660; doi:10.1038/s41467-023-37435-7)
Supplement: Supplementary file 1 — Supplementary Information [file 41467_2023_37435_MOESM1_ESM.pdf]

Supplementary information of

Future Indian Ocean warming patterns

Sahil Sharma[1,2], Kyung-Ja Ha[1,2,3*], Ryohei Yamaguchi[4], Keith B. Rodgers[1,5]
Axel Timmermann[1,5], Eui-Seok Chung[6]

1. Center for Climate Physics, Institute of Basic Science, Busan, South Korea
2. Department of Climate System, Pusan National University, Busan, South Korea
3. BK21 School of Earth and Environmental Systems, Pusan National University, Busan, South Korea
4. Japan Agency for Marine-Earth Science and Technology, Yokosuka, Japan
5. Pusan National University, Busan, South Korea
6. Division of Atmospheric Sciences, Korea Polar Research Institute, Incheon, South Korea

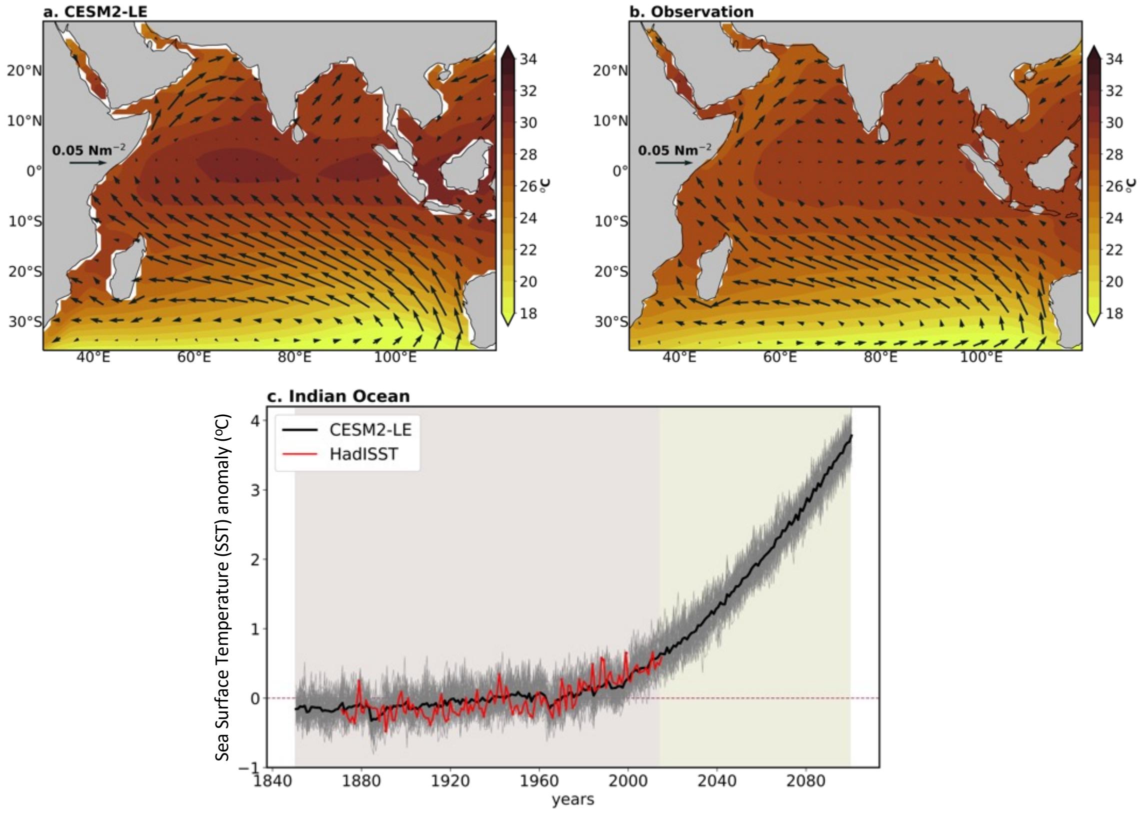

Supplementary Figure 1. Comparison of Observations with model simulations. (a) Ensemble mean climatology of sea surface temperature (SST) (ºC, shaded) and surface wind stress (N/m^2, vectors) for the 50 members of CESM2-LE during the historical period (1980-2000). **(b)** is same as in **(a)** but for observation using HadISST (SST) and SODA (wind stress). **(c)** Time series of the area-averaged SST anomaly (black, ºC) over the Indian Ocean (IO) for ensemble members (thin) and ensemble mean (thick) using the CESM2-LE simulations during the time-period 1850-2100. The red color time series represents the area-averaged SST anomaly over the IO using the HadISST observation dataset during the time-period 1870-2015. All the time series are relative to the 1870-2014 baseline.

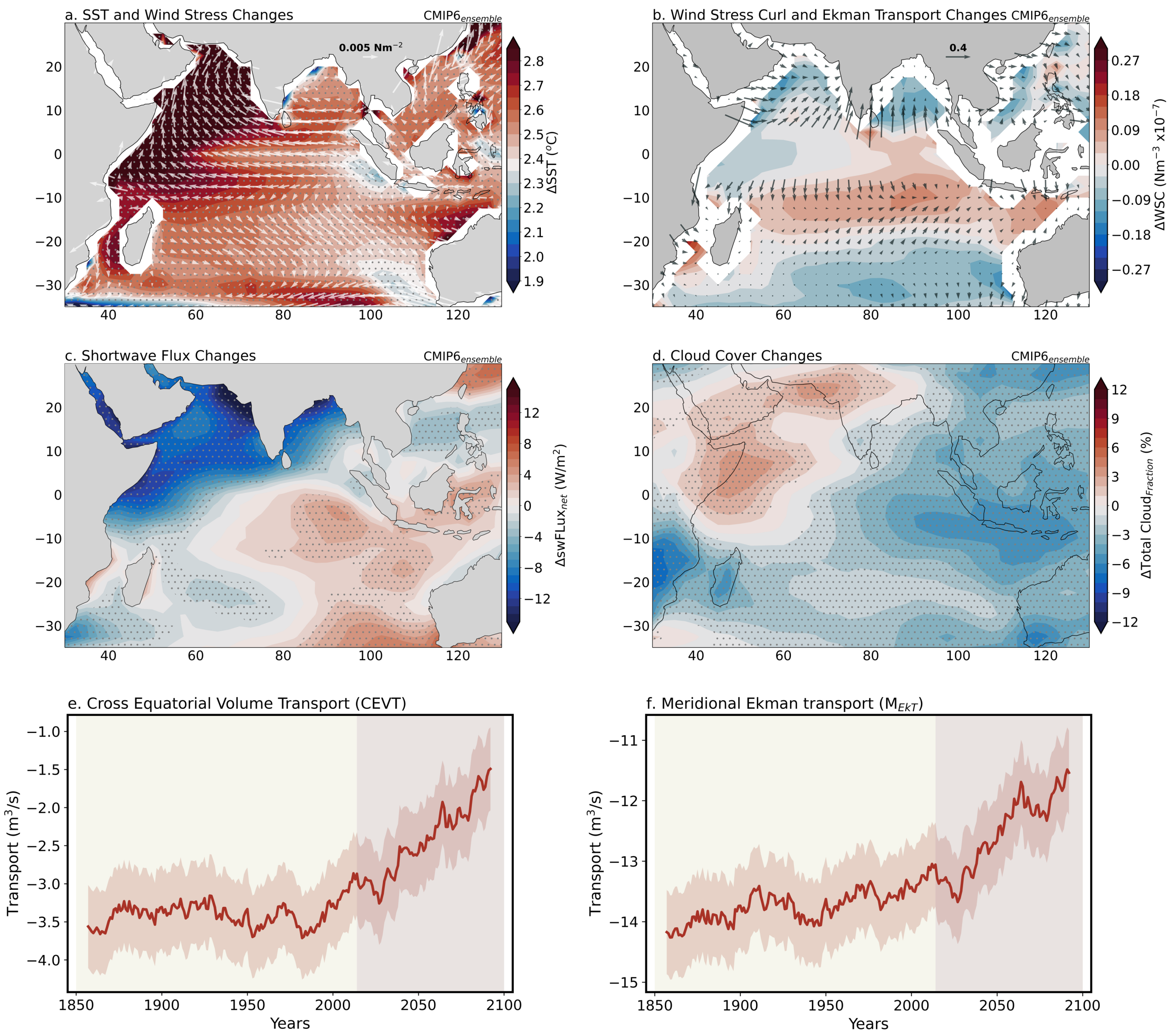

Supplementary Figure 2. Drivers of warming pattern in the Indian Ocean in CMIP6 model simulations. Ensemble mean changes in **(a)** SST (shaded) and surface wind stress (vectors) **(b)** Wind stress curl (shaded) and Ekman transport (vectors) **(c)** short wave radiative flux and **(d)** total cloud cover for CMIP6 (13 models). The ensemble mean changes are calculated by taking the difference between the future (2080-2100) and historical (1980-2000) period in all the simulations. Stippling in (a-d) indicate the regions where the difference between future and historical changes is significant at 95% confidence level based on a two-sample t-test on the time series of ensemble means. The bottom panel show the ensemble mean area averaged time series of **(e)** cross-equatorial volume transport (CEVT) over the equatorial western Indian Ocean (WIO), and **(f)** meridional Ekman transport (M_{EkT}) over the Arabian sea (AS). See Figure 4 for the locations used to calculate the area-averaged of the various quantities. The dark shading across the time series represents the one ensemble standard deviation.

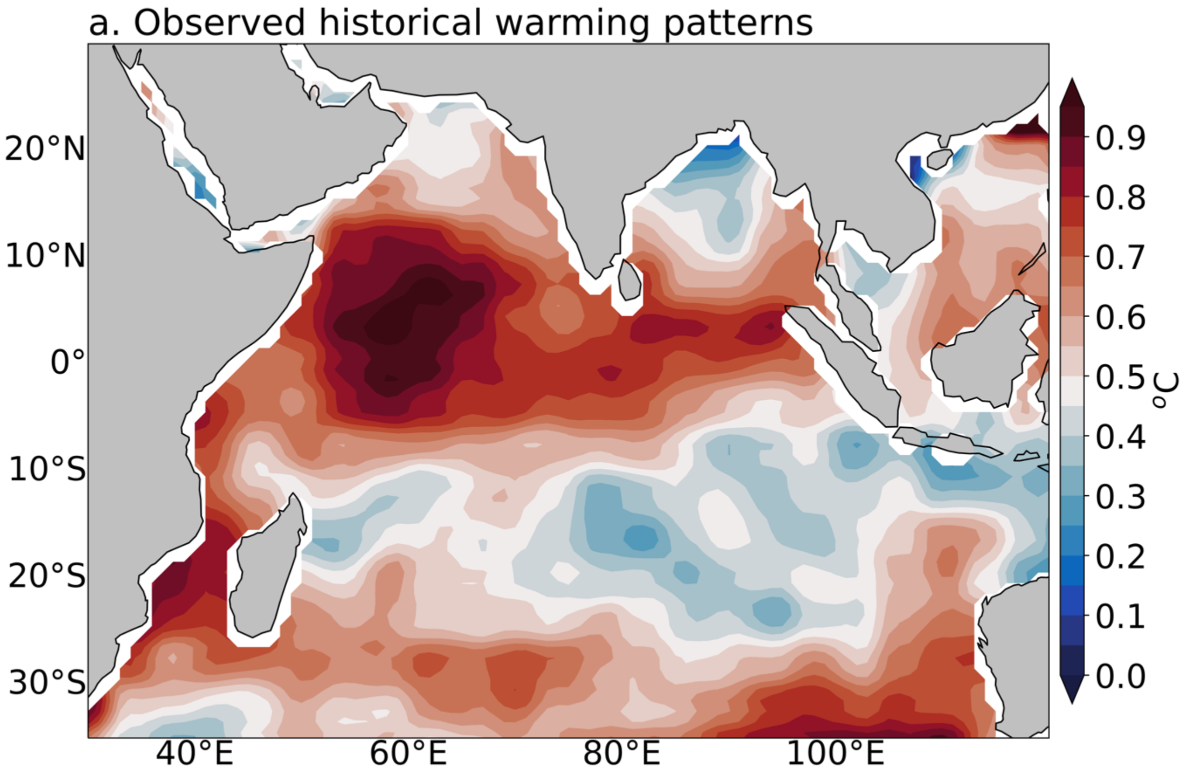

Supplementary Figure 3. Historical Indian Ocean warming patterns. (a) Observed historical SST changes based on the HadISST dataset. The changes are calculated by taking the difference between the two periods (1880-1920 minus 1980-2020).

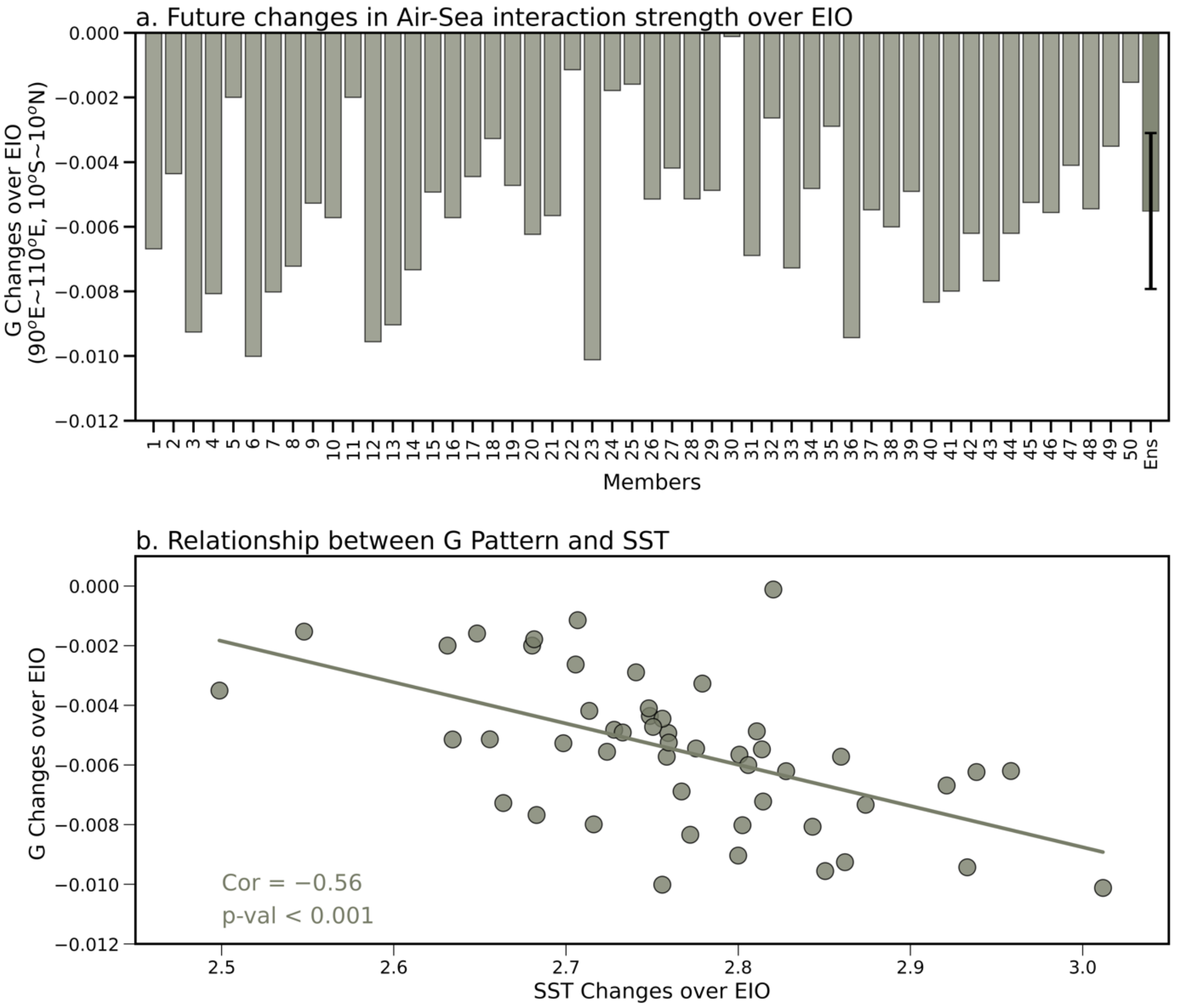

Supplementary Figure 4. Future changes in G pattern and its relationship with SST over eastern Indian Ocean (EIO). (a) The bar plot shows the future changes (future minus historical) in *G* over the EIO (90ºE:110ºE, 10ºS:10ºN) for 50 ensemble member and ensemble mean. The negative value represents the strong damping of SST anomalies. The error bars show the ensemble standard deviation. **(b)** Scatter plot shows the relationship between the *G* and SST changes over the EIO. Each dot in the scatter plot shows the ensemble member and the line represents the slope across the ensemble members over the different region. Correlation coefficients and corresponding *P* values are indicated. The relationship between the future changes in *G* and SST over the EIO, indicating a significant negative correlation, suggests that negative feedback of atmospheric damping on SST anomalies will enhance in the future climate.

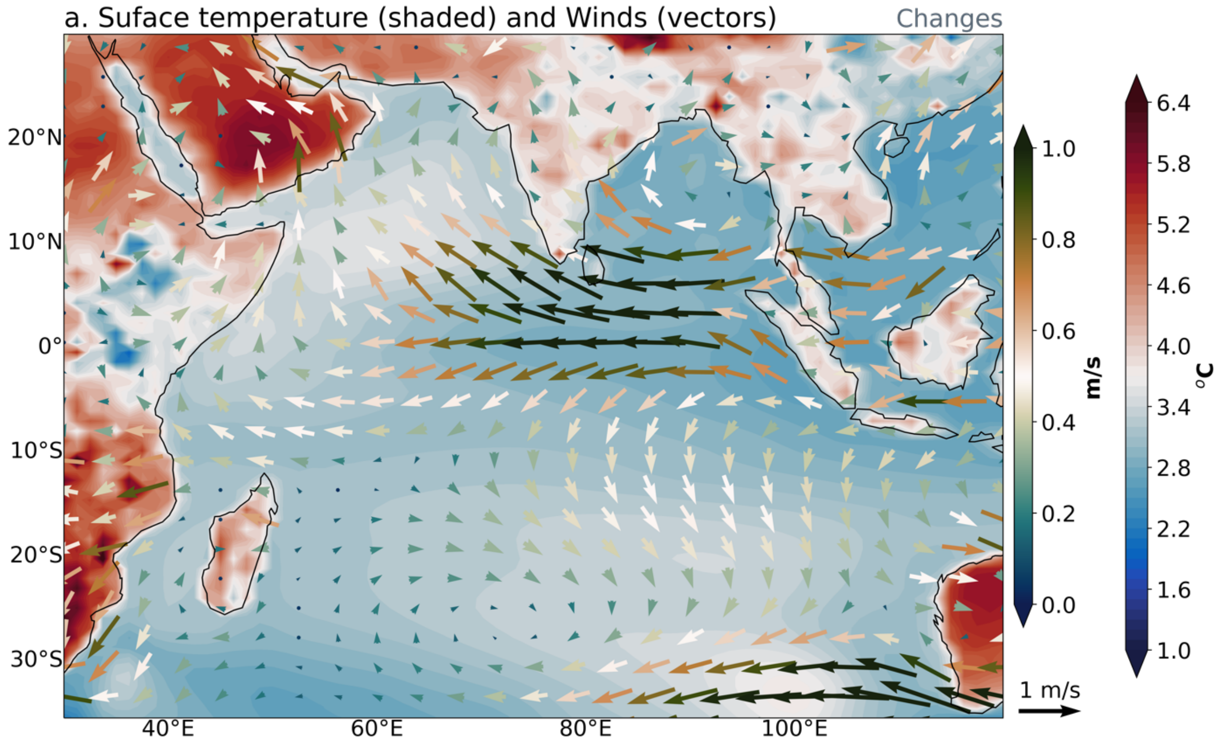

Supplementary Figure 5. Surface temperature and surface wind changes in the CESM2-LE simulations. Ensemble mean changes of **(a)** surface temperature (shaded) and surface winds (vectors) for the 50 ensemble members of CESM2-LE. The mean state changes are calculated by taking the ensemble mean difference between the future (2080-2100) and the historical (1980-2000) periods. The color vectors represent the magnitude of the surface winds.

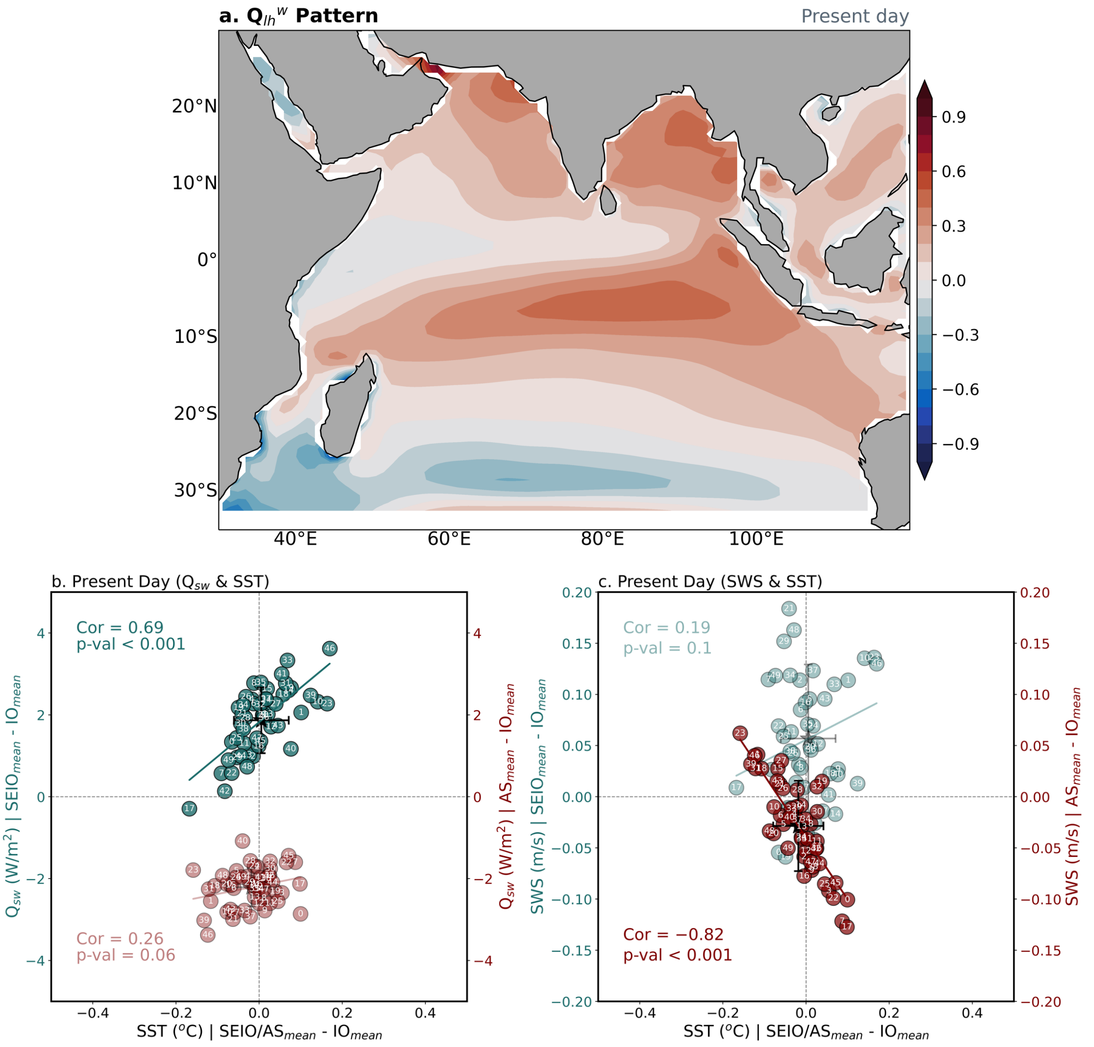

Supplementary Figure 6. Present-day atmospheric forcing adjustment processes in the Indian Ocean. Ensemble mean of **(a)** Atmospheric wind effect on the latent heat flux ($Q_{lh}{}^{w}$ $=<\overline{Q_{lh}}\times\acute{W}/\overline{W}>$where $\overline{Q_{lh}}$ and $\overline{W}$ is the climatological latent heat flux and surface wind speed during the period 1980-2000 and $\acute{W}$ is perturbation of surface wind speed from the mean and the brackets <…> represents the ensemble mean). **(b)** The scatter plot represents the relationship between shortwave radiative flux (Q_{sw}) and SST over the Arabian Sea (AS) (red) and southeastern Indian Ocean (SEIO) (green). Note that IO mean removed from each area-averaged region. Each dot in the scatter plot shows the ensemble member and the black star in the middle represents the ensemble mean. Also, the red and green line represents the slope across the ensemble members over the different regions. **(c)** is same as in (b) but shows the relationship between surface wind speed (SWS) and SST, respectively. Correlation coefficients and corresponding P values are indicated.

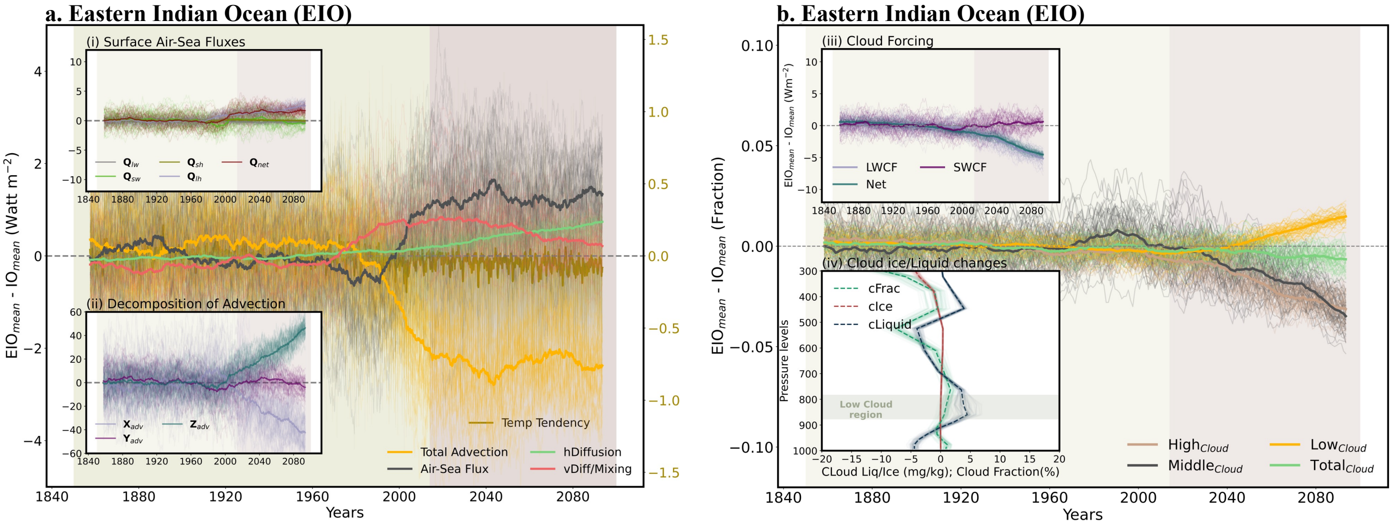

Supplementary Figure 7. Contribution of different factors to the reduced degree of warming over the Eastern Indian Ocean (EIO). Time series of each term of the vertically-integrated (0-65m) ocean heat budget equation over **(a)** the EIO (85°E:105°E, 10°S:5°N) from 1850-2100 for 50 ensemble members of the CESM2-LE. Inset plot **(i)** in **(a)** shows the time series of each component of the surface air-sea flux (positive values represent anomalous heat input into the ocean) components including shortwave radiation (Q_{sw}), longwave radiation (Q_{lw}), sensible heat flux (Q_{sh}), latent heat flux (Q_{lh}), and net surface air-sea flux (Q_{net}) and **(ii)** represents the time series of vertically integrated individual advection terms such as zonal (X_{adv}), meridional (Y_{adv}) and vertical (Z_{adv}) advection. **(b)** Time series of vertically-integrated cloud cover over the EIO from 1850-2100 for 50 ensemble members. Inset plot **(iii)** in **(b)** shows the time series of the longwave cloud forcing (LWCF), shortwave cloud forcing (SWCF), and net (LWCF+ SWCF) cloud forcing, and **(iv)** represents the changes in cloud liquid amount, cloud ice amount and cloud fraction over the EIO from the surface to 300hPa. Note that the IO mean is removed from each area-averaged time series over the EIO. All the time series are relative to an 1850-2014 baseline, and finally, 15-year running means are calculated. Thick (thin) lines represent the ensemble mean (individual ensemble members) in all the plots.

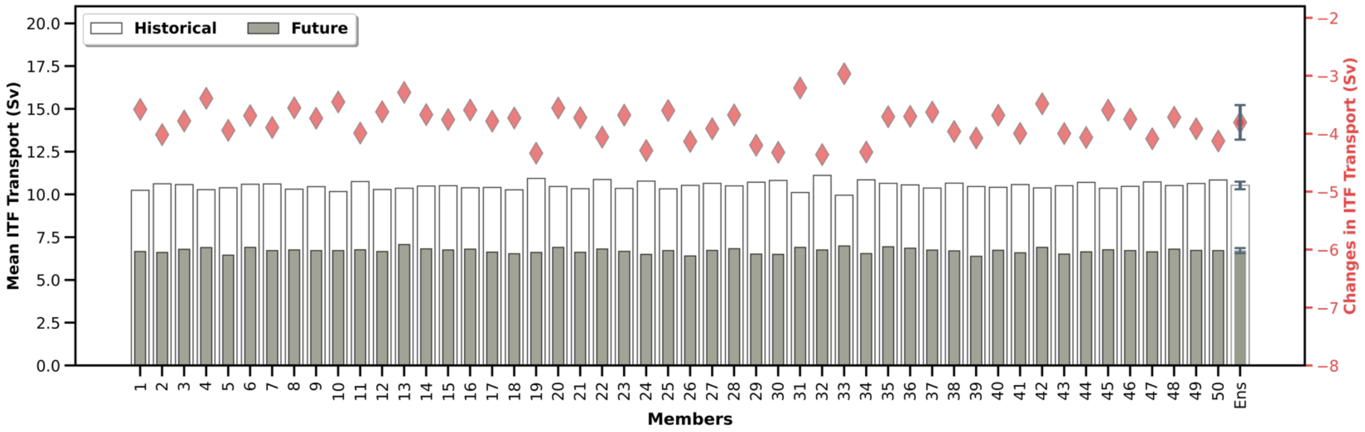

Supplementary Figure 8. Projected weakening of Indonesian throughflow (ITF). The white (gray) bars show the mean ITF transport for the 50 ensemble members and the ensemble mean during the historical (future) period. Also, the red diamond markers show the changes (Future minus historical) across each ensemble member and ensemble mean. The negative value represents the reduction in transport from the Pacific to the Indian Ocean. The error bars show the ensemble standard deviation. The ITF transport is estimated by taking the barotropic stream function difference between 10°S and 20°S, along the 115°E meridional section.

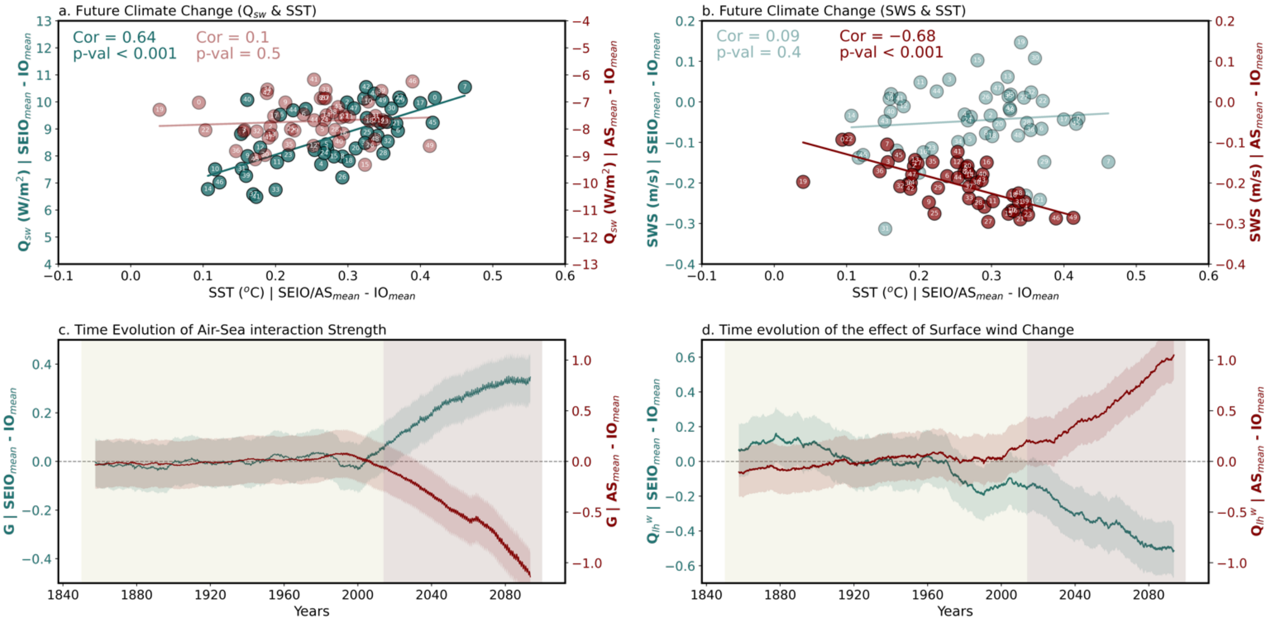

Supplementary Figure 9. Relationship of SST with other factors over AS and SEIO in the future. The scatter plot represents the **(a)** projected change (future minus historical) relationship between Q_{sw} and SST over the AS (red) and SEIO (green). Note that IO mean removed from each area-averaged region. Each dot in the scatter plot shows the ensemble member and the red/green line represents the slope across the ensemble members over the different region. **(b)** are same as in (a) but shows the future relationship between surface wind speed (SWS) and SST. **(c)** Ensemble mean time series of the monthly air-sea interaction strength (*G*) over AS and SEIO during the time period 1850-2100. The *G* is multiplied by a factor of 10^3. Note that IO mean is removed from each area-averaged time series over the different region. Both the time series are relative to the 1850-2014 baseline, and finally, the 15-years running mean is calculated. The shading represents the ± 1 standard deviation across the ensemble members. **(d)** same as in (c) but for atmospheric wind effect on the latent heat flux (Q_{lh}^w).

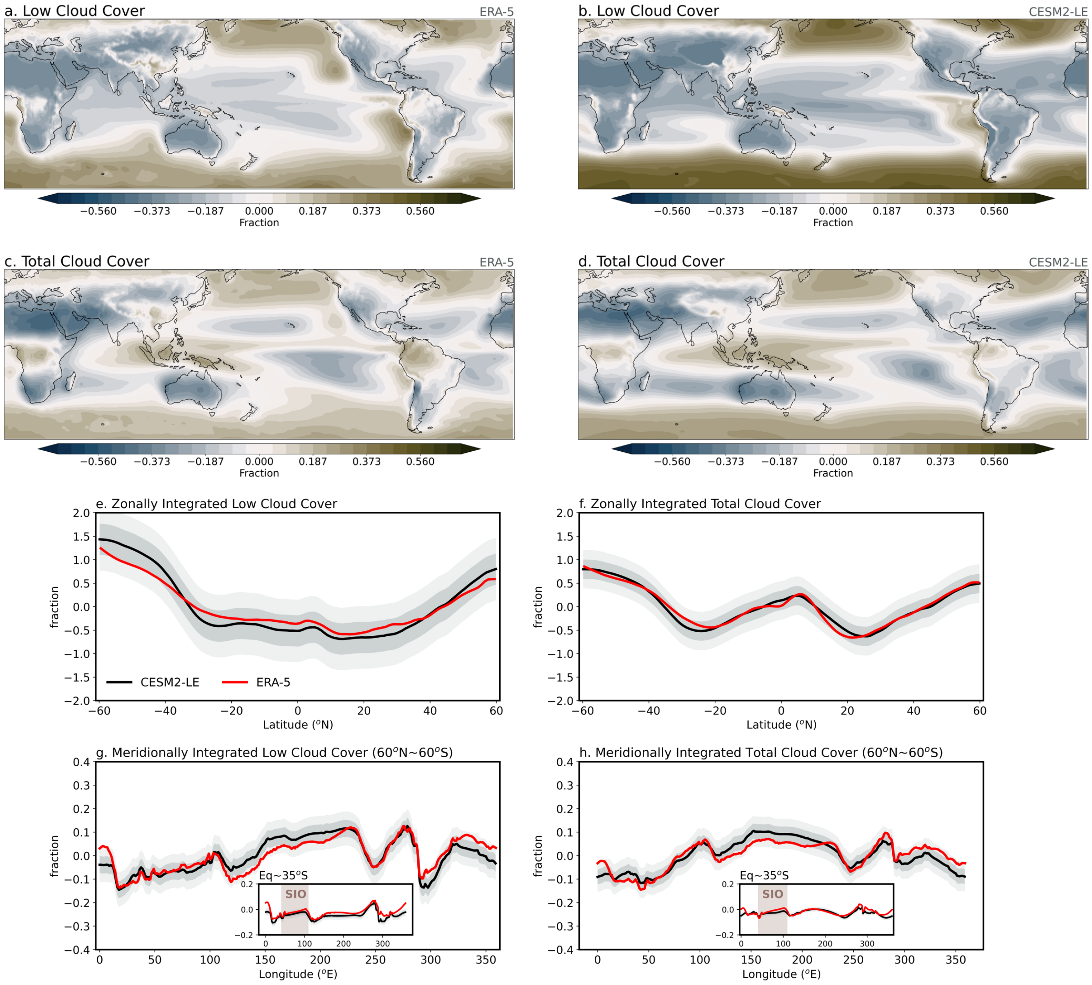

Supplementary Figure 10. Comparison of vertically integrated low and total cloud cover in the ERA-5 and CESM2-LE simulations. (a) Climatology of vertically integrated low-cloud cover (in fraction) during the time period 1980-2020 in ERA-5 observational dataset. **(b)** Ensemble mean climatology of vertically integrated low-cloud cover (in fraction) during the time period 1980-2020 for the 50 ensemble members in CESM2-LE simulation. Note that the in both (a) and (b) 60°N~60°S mean values are removed from the climatological values. **(c, d)** are same as (a, b) but for vertically integrated total cloud cover. **(e)** Zonally integrated (0E~360°E) low-cloud cover in ERA-5 (red) and ensemble mean in CESM2-LE (black). **(f)** same as (e) but for the total-cloud cover. **(g)** Meridionally integrated (60°N~60°S) low-cloud cover in ERA-5 (red) and ensemble mean in CESM2-LE (black). The inset plot shows the meridionally integrated low-cloud cover in the southern hemisphere (equator~35°S) only. The region of southern Indian Ocean (40°E~110°E) is highlighted with light red shading. **(h)** same as (g) but for the total-cloud cover. The dark (light) grey shading across the line in (e, f, g and h) represents the 0.5 (1) ensemble standard deviation.

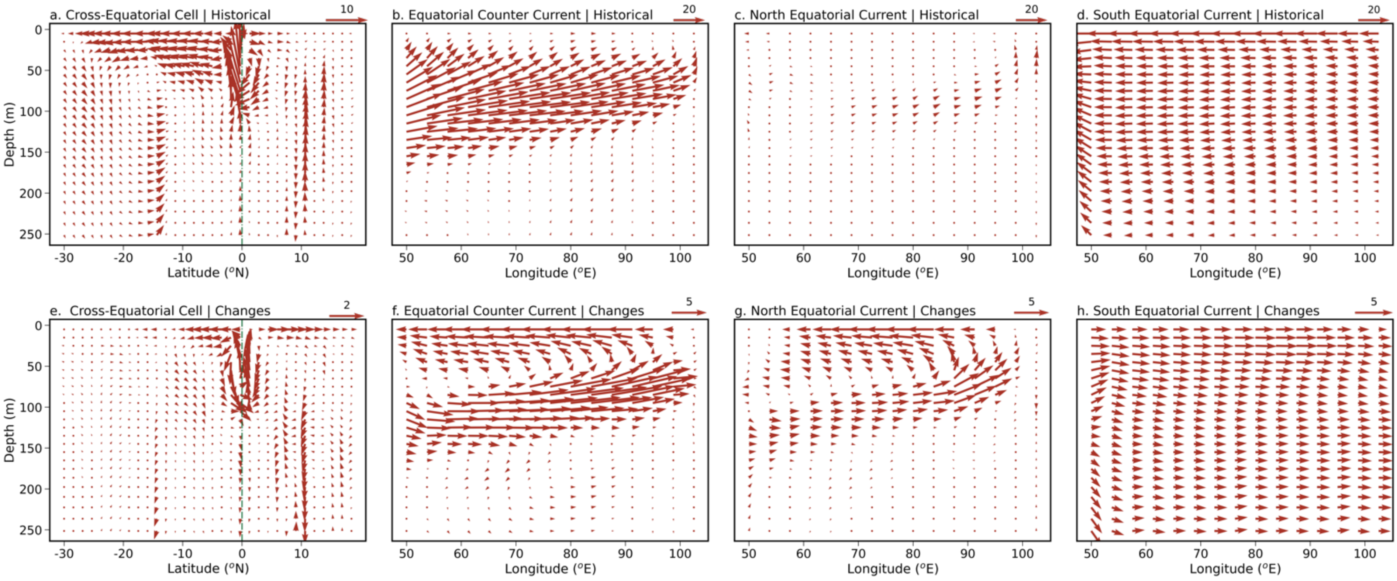

Supplementary Figure 11. Ocean Currents climatology and changes in the Indian Ocean in CESM2-LE simulations. Upper panel shows the ensemble mean climatology of **(a)** cross-equatorial cell (product of meridional velocity and vertical velocity averaged over the region 55°E:65°E), **(b)** Equatorial Counter Current (product of zonal velocity and vertical velocity averaged over the region 2°S:2°N), **(c)** North Equatorial Current (product of zonal velocity and vertical velocity averaged over the region 2°N:5°N), and **(d)** South Equatorial Current (product of zonal velocity and vertical velocity averaged over the region 15°S:8°S) for the 50 members of CESM2-LE during the historical period (1980-2000). (**e, f, g and h)** in the lower panel is same as in **(a, b, c and d)** but for the ensemble mean changes by taking the difference between the future (2080-2100) and the historical (1980-200) periods.

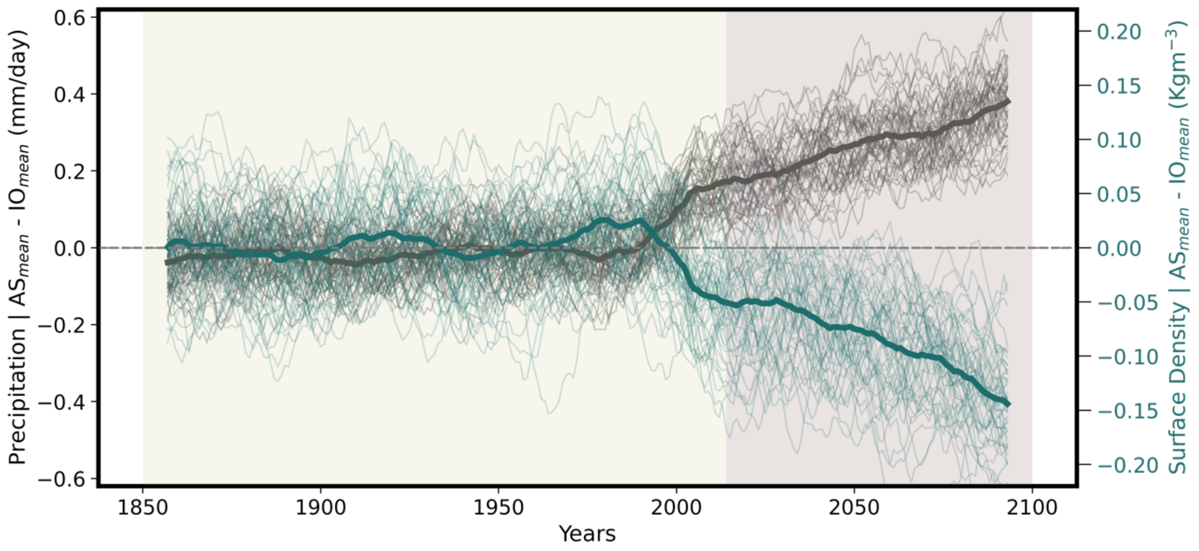

Supplementary Figure 12. Time evolution of precipitation and surface density changes over the Arabian Sea. Time series of the precipitation (black, mm/day) and surface density (green, Kg/m^3) from 1850-2100 for 50 ensemble members. Note that IO mean is removed from each area-averaged time series over the AS. All the time series are relative to the 1850-2014 baseline, and finally, the 15-years running mean is calculated. Thick (thin) lines represent the ensemble mean (ensemble member) in all the plots.

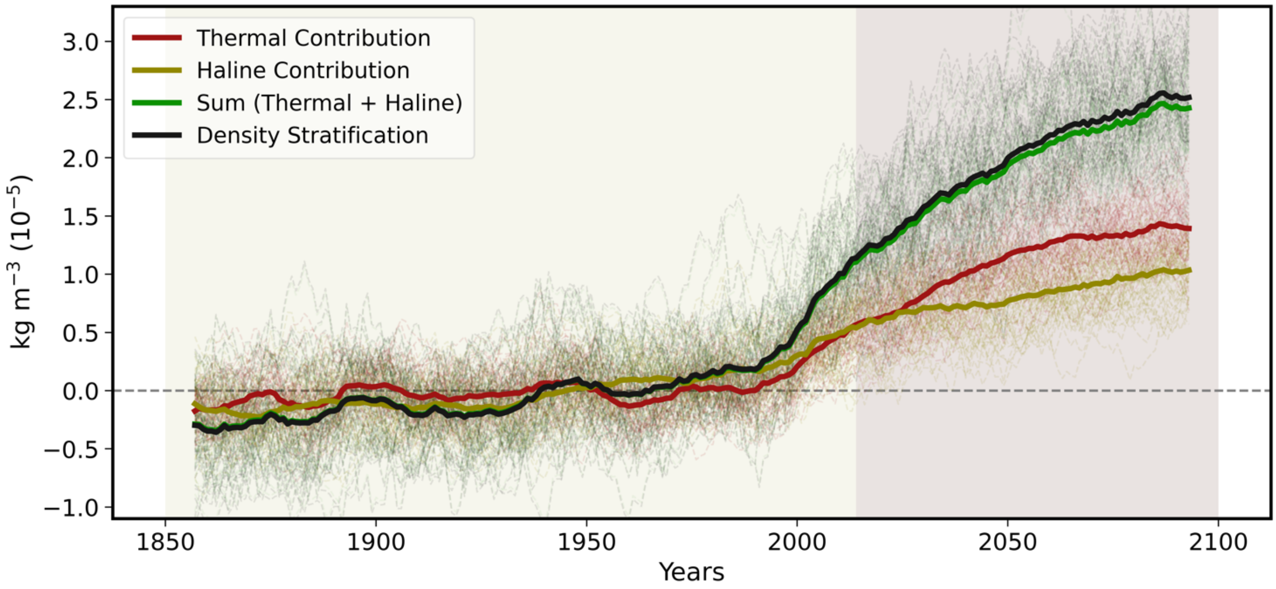

Supplementary Figure 13. Time evolution of stratification changes in the Arabian Sea. Time series of the vertical density gradient, i.e., density stratification (black) at a depth of 65m. The density stratification is calculated using the following equation: $\Delta\rho = \alpha\Delta T + \beta\Delta S$ (see methods for more details). Also, the thermal (red) and haline (brown) contribution to the density stratification is indicated. The sum of thermal and haline changes is almost equal to the density changes, although there are some errors due to non-linear contribution. All the time series are relative to 1850-2014 baseline. A 15-year running mean is applied to all the time-series. Thick (thin) lines represent the ensemble mean (ensemble member) in all the plots.

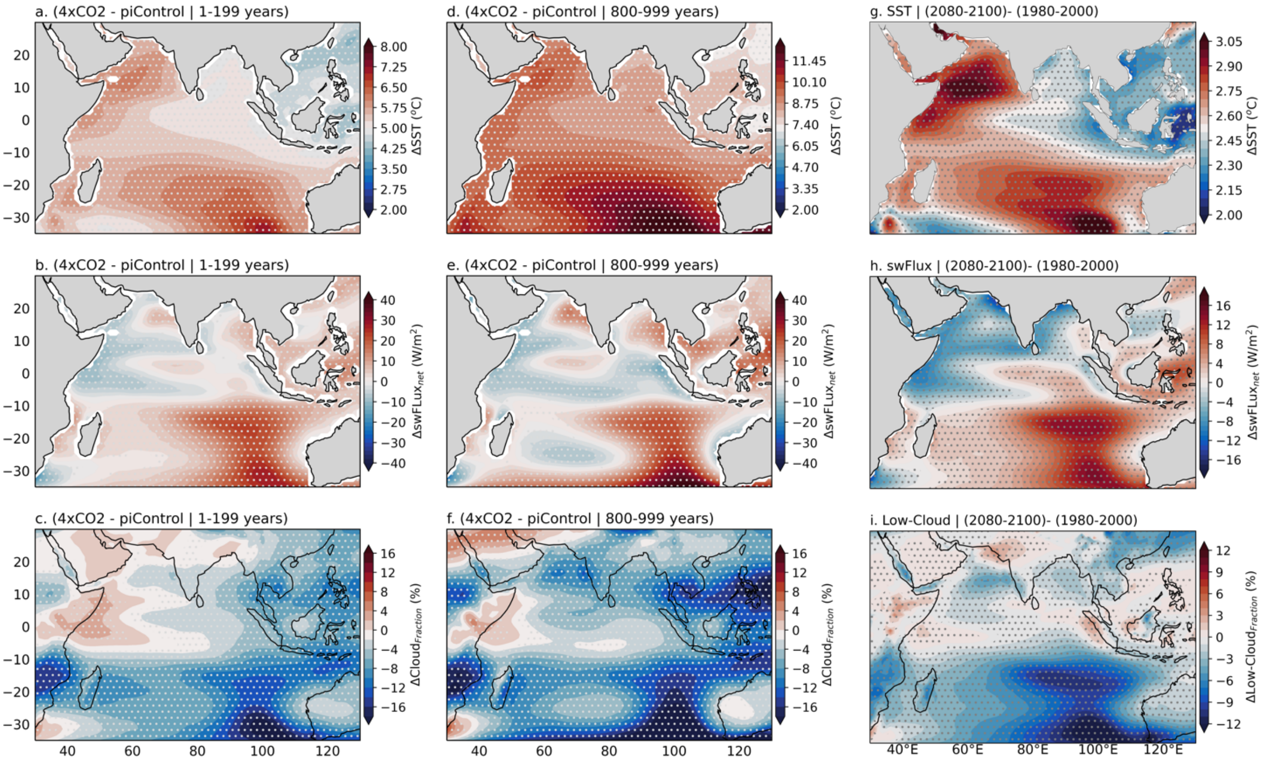

Supplementary Figure 14. Comparison of CESM2-LE with CMIP6-CESM2 simulations. Left panel shows the difference of abrupt 4xCO_2 minus pre-industrial control of **(a)** SST (°C), **(b)** shortwave flux (W/m^2), and **(c)** cloud fraction (%) for the first 200 years of the experiment in CMIP6-CESM2. (**d, e, and f)** in the middle panel are same as in (**a, b, and c)** but for the last 200 years. Stippling in the left and middle panels indicates the regions where the difference between the abrupt 4xCO_2 and piControl is significant at 95% confidence level based on a two-sample t-test. Right panel shows the ensemble mean changes in **(g)** SST (°C), **(h)** shortwave flux (W/m^2), and **(i)** cloud fraction (%) for the first 50 members of CESM2-LE. The mean state changes are calculated by taking the difference between the future (2080-2100) and the historical (1980-2000) periods in all the ensemble members. Stippling indicates the regions where the difference between future and historical changes is significant at 95% confidence level based on a two-sample t-test on the time series of ensemble means.

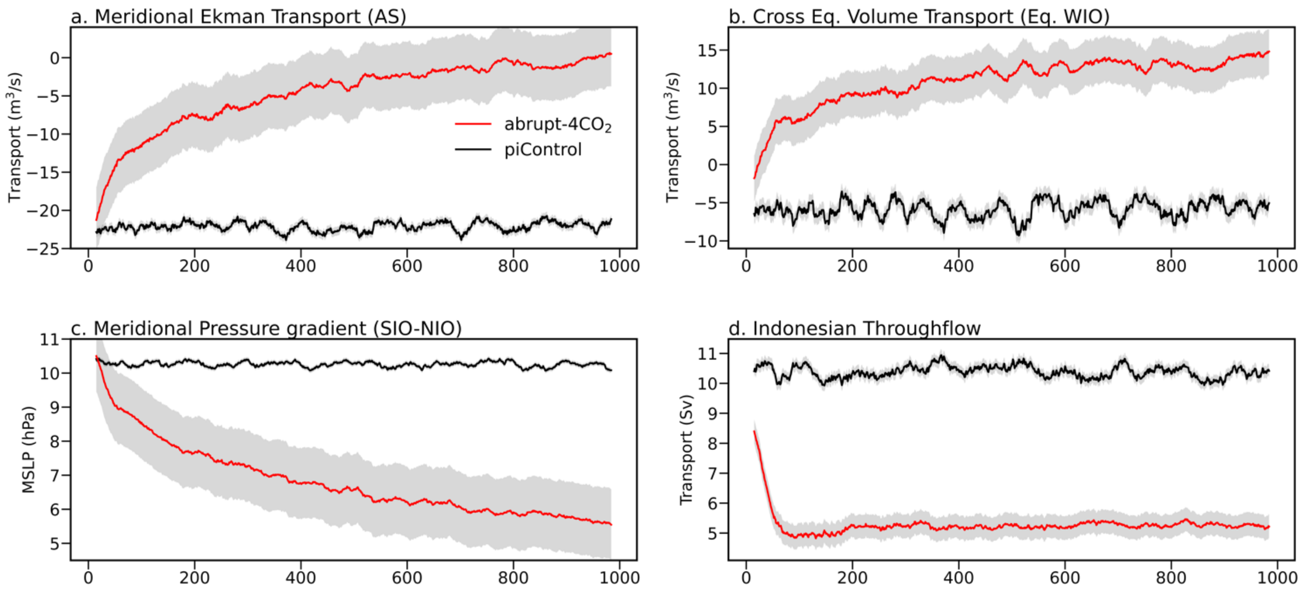

Supplementary Figure 15. Temporal evolution of various quantities in CMIP6-CESM2 experiments. Temporal evolution of abrupt-4xCO2 (red) and pre-industrial control (black) of (**a**) meridional Ekman transport over the AS, **(b)** cross-equatorial volume transport over equatorial WIO, **(c)** meridional pressure gradient (difference between southern Indian Ocean (10S:35S, 40E:100E) and northern Indian Ocean (10N:30N, 40E:100E), and **(d)** Indonesian throughflow. See Figure 4 for the locations used to calculate the area-averaged of the various quantities. A 30-year running mean is applied to each time series, and the shading indicated one standard deviation. Less negative values in (a and b) show the weakening of southward transport. Lower positive values in (c and d) indicate the weakening of meridional pressure gradient and Indonesian throughflow, respectively.

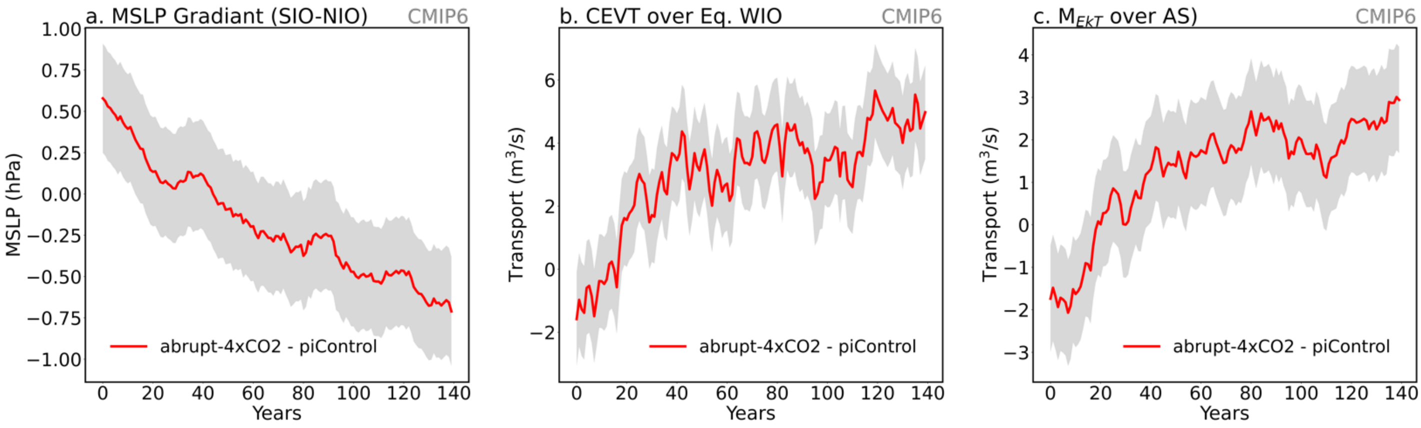

Supplementary Figure 16. Temporal evolution of pertinent variables showing the difference between CMIP6 abrupt-4xCO2 and piControl experiments. Temporal evolution of the difference between abrupt-4xCO2 and pre-industrial Control simulation of **(a)** meridional pressure gradient (difference between southern Indian Ocean (10°S-35°S, 40°E-100°E) and northern Indian Ocean (10°N-30°N, 40°E-100°E), **(b)** cross-equatorial volume transport over equatorial WIO, and **(c)** meridional Ekman transport over the AS. An 11-year running mean is applied to each time series, and the shading indicates one standard deviation. Less negative values in (b and c) indicate a weakening of southward transport. The CMIP6 model used to calculate the multimodal ensemble mean are BCC-CSM2-MR, CESM2-FV2, CMCC-ESM2, MCM-UA-1-0, MRI-ESM2-0, TaiESM1, UKESM1-0-LL, UKESM1-1-LL.

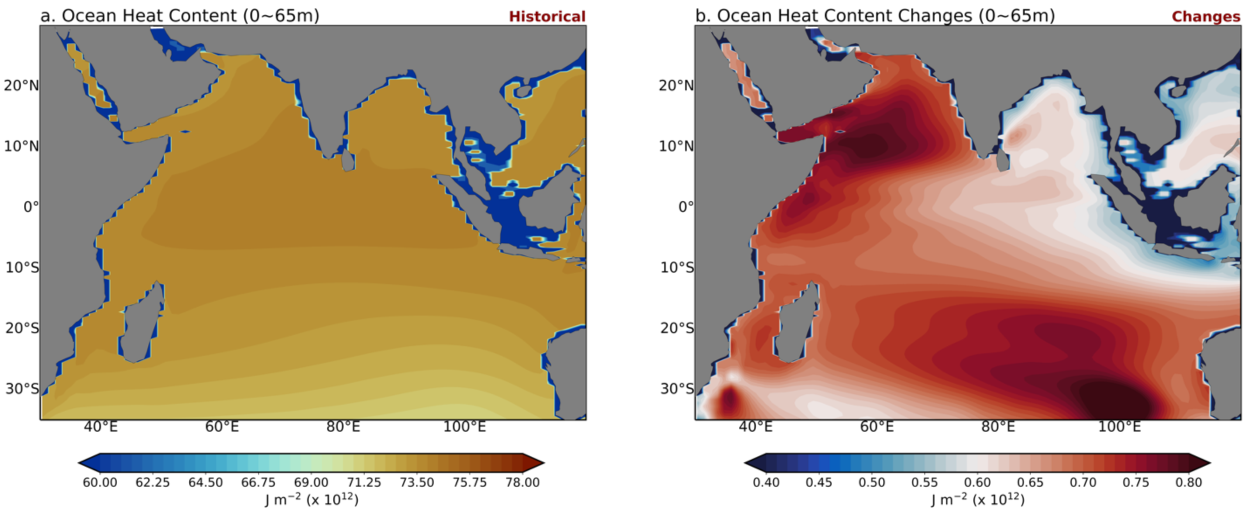

Supplementary Figure 17. Climatology and changes of ocean heat content in the upper 65m in CESM2-LE simulations. (a) Ensemble mean climatology of ocean heat content for the 50 members of CESM2-LE during the historical period (1980-2000). **(b)** Ensemble mean changes of ocean heat content. The changes are calculated by taking the difference between the future (2080-2100) and historical (1980-2000) periods. Ocean heat content is calculated as: $\mathrm{OHC} = c_p \int_{-65}^{0} \rho(z)T(z)dz$, where c_p is the specific heat capacity of water, $\rho(z)$ is the sea water density profile and T(z) is the temperature profile.
